# Pilot Study of a Faith-Based Physical Activity Program Among Sedentary Blacks

**Published:** 2008-03-15

**Authors:** Melicia C Whitt-Glover, Patricia E Hogan, Wei Lang, Daniel P Heil

**Affiliations:** Department of Epidemiology and Prevention, Division of Public Health Sciences, Wake Forest University Health Sciences; Department of Biostatistical Sciences, Division of Public Health Sciences, Wake Forest University School of Medicine, Winston-Salem, North Carolina; Department of Biostatistical Sciences, Division of Public Health Sciences, Wake Forest University School of Medicine, Winston-Salem, North Carolina; Department of Health and Human Development, Montana State University, Bozeman, Montana

## Abstract

**Introduction:**

Physical activity participation is low among blacks, and strategies are needed to successfully create immediate and sustained behavior change related to physical activity. Churches can play an important role in health promotion efforts among blacks because of their central role in spiritual guidance, communication, social support, and networking. This pilot study evaluated the feasibility and acceptability of implementing a physical activity program for sedentary black adults in churches.

**Methods:**

We used a preintervention/postintervention single-group design to evaluate the effect of a 3-month faith-based physical activity intervention on daily walking and moderate- and vigorous-intensity physical activity among sedentary blacks. Eighty-seven black adults participated in eight group sessions that included discussion of physical activity-related topics, an instructor-led physical activity session, and weekly incentives to promote physical activity. We used a questionnaire to assess moderate and vigorous physical activity in minutes per week at baseline and after 3 months. Walking was assessed weekly in steps per day by using a pedometer.

**Results:**

Participants (mean age, 52 yrs; mean body mass index, 35 kg/m^2^) reported 27 ± 54 and 10 ± 25 minutes per week in moderate-intensity and vigorous-intensity physical activity, respectively, and walked 4822 ± 2351 steps per day at baseline. After 12 weeks, moderate- and vigorous-intensity physical activity increased by 67 ± 78 and 44 ± 66 minutes per week, respectively (*P* ≤ .01), and daily walking increased by 1373 ± 728 steps per day (*P* < .001).

**Conclusion:**

These data suggest that a faith-based physical activity intervention may be an appropriate strategy for increasing physical activity among sedentary black adults. Future research will determine the impact of this program in a randomized, controlled design.

## Introduction

The benefits of increased physical activity (PA) have been well documented (1-4), yet national data show that only 24% to 36% of black adults (aged 18 years or older) reported participating in regular physical activity (5). Churches may be an ideal setting for health promotion efforts in black communities because of their central role in spiritual guidance, communication, social support, and networking (6-9). Faith-based interventions among blacks have been successful for smoking cessation (10), reducing cardiovascular disease risk factors (11-13), and increasing fruit and vegetable consumption (14-16). Several faith-based studies among blacks have included PA within programs focusing on other outcomes (6,17-21). These studies showed increases in PA levels, but most showed no significant differences between intervention and control participants. One limitation of previous faith-based studies may have been limited attention to incorporating tenets of the faith-based organization (e.g., religious beliefs, scriptural references) into intervention strategies and limited involvement of the faith-based organization in intervention development. These factors may play an important role in determining intervention success, but additional research is needed to develop interventions that address these factors.

The purpose of this study was to develop and test a faith-based PA program to increase participation in daily walking and moderate- and vigorous-intensity physical activity (MPA and VPA, respectively), particularly in bouts lasting 10 minutes or longer, among sedentary black adults. This study was funded as a part of a career development grant. Data collected during this study were intended to serve as pilot data for a proposal for a larger randomized, controlled trial to test the faith-based PA program compared with a control group.

## Methods

### Study design

We conducted this study in a suburban community in North Carolina from March through October 2005. In September and October 2004, nine local church pastors participated in in-depth interviews and provided input on the design, development, and implementation strategies for health promotion programs in churches in general and a faith-based PA program specifically. Pastors were recruited from a list of pastors who attended (or sent a representative to) at least one of two luncheons held for ministers in the local community to determine potential strategies for health promotion and disease prevention among blacks.

Data from in-depth interviews suggested that, in general, health was viewed as a concept that encompassed spiritual, physical, mental, and emotional health. Pastors indicated that they play a role in the health of their church congregants by setting an example for healthy living, imparting the knowledge that wholeness and health are essential parts of Christian character that enable one to do God's bidding and that the body is God's temple. They advocate, encourage, and support efforts to engage in healthy behaviors and to implement health-related programming within the church. Pastors provided suggestions for why their congregants were not physically active (e.g., lack of motivation, knowledge, time, or resources) as well as feedback on ways to successfully implement a faith-based intervention (e.g., getting buy-in and support from the pastor, making sure to offer sessions during times that do not conflict with other church activities, linking health-promoting activities to church ministries rather than creating separate programs).

We used information from the in-depth interviews to shape the study design and session content of the faith-based PA intervention. Because the pilot study was designed to determine the feasibility and acceptability of the intervention strategy, a preintervention and postintervention study design with no control group was used. This design was deemed the most acceptable for church pastors and is in line with previous research suggesting that a no- or low-attention control would not be well received by this population (22). The intervention, which was based on social cognitive theory (23,24), included eight weekly group sessions focusing on behavioral strategies to increase daily MPA and VPA. Weekly sessions included 30 minutes of MPA and a 60-minute discussion session (Table 1). Sessions opened and closed with prayer, and session content was presented from a theological perspective with a focus on personal health care as a method of protecting God's temple. Session five focused entirely on healthful living and negotiating barriers from a Biblical perspective. Participants discussed scriptural references that supported the notion of self-care and negotiating barriers, which were used throughout the remaining sessions. Incentive items were provided at each session to encourage PA participation. A certified fitness instructor conducted the 30-minute MPA session and provided an opportunity for participants to practice using incentive items. Incentives were culturally relevant items such as faith-based aerobics videos, a gospel exercise CD, a tote bag, and a T-shirt with a faith-based slogan that fit the intervention theme.

Participants maintained weekly logs of pedometer step counts, which were used to self-monitor walking, and received a weekly summary to track walking progress throughout the study. We used incentives to encourage participants to increase their daily MPA to at least 30 minutes by engaging in moderate-intensity walking. Although participants were not given specific targets to increase their steps per day, they were advised of the 10,000 steps recommendation for daily walking (25) and were told that striving to reach this goal was in line with achieving the recommendation for MPA.

We hired group leaders to lead the weekly intervention sessions at churches. The following characteristics were considered desirable for group leaders: 1) having a working knowledge of general health and wellness but not being currently employed as a health educator, 2) being physically active but not exceptionally athletic, 3) having previous experience working with blacks and other groups in faith-based settings, 4) being comfortable speaking in group settings; and 5) not being a member of a church involved in the intervention. These criteria were selected to avoid hiring highly trained individuals, which might hamper the ability to disseminate this type of intervention to a larger audience, and to avoid potential biases by hiring church members because all four churches could not be represented in group leadership. Two black females were recruited as group leaders. Each group leader was assigned to lead sessions at two churches, depending on the time the church selected to hold the sessions and the availability of the group leader.

Each church pastor also selected one church member to serve as a liaison between the church and the study staff and to attend to any church-related logistical issues. We paid church liaisons a modest honorarium for their participation in the study. In addition, church liaisons received a group leader training manual and session materials to facilitate continued implementation of the PA program after completion of the study. No additional incentives or compensation were provided to churches or pastors.

### Study participants

We selected a convenience sample of four churches that identified themselves as predominantly serving blacks and whose pastors participated in one of the previously mentioned ministers’ luncheons. Recruitment for study participants was at the churches’ discretion; it primarily consisted of making announcements during Sunday morning worship services and weekly activities and placing flyers throughout the church and in the Sunday bulletin. Interested individuals were invited to attend a general-interest session held at each church and led by the study’s principal investigator (MCW). Interest sessions were held during the time the church had selected for the weekly intervention sessions. This limited the number of individuals who would be unable to attend the sessions regularly. During the interest session, the principal investigator explained the study procedures and eligibility criteria and answered participant questions. At the end of the interest session, participants were screened for study eligibility using the following criteria: 1) being self-identified as black; 2) being 18 years of age or older; 3) not currently meeting recommendations for MPA or VPA (defined as self-report of 30 minutes or less of MPA on 5 or fewer days per week; 20 minutes or less of VPA on 3 or fewer days per week, *or* obtaining recommended MPA and VPA, but in bouts lasting less than 10 minutes, as assessed using a modified version of the International Physical Activity Questionnaire); 4) responding “no” to all questions on the Physical Activity Readiness Questionnaire (PAR-Q) *or* having obtained medical clearance; 5) having no other physical illnesses or disabilities limiting PA; and 6) being willing to commit to participating in weekly intervention sessions and all data collection visits. All individuals who attended an interest session received a pedometer, regardless of their eligibility for study participation. Eighty-seven participants met the eligibility criteria and were enrolled in the study.

All study procedures were approved by the Institutional Review Board at Wake Forest University Health Sciences. Eligible participants provided written informed consent before participating in data collection or in the study intervention.

### Intervention fidelity

Intervention fidelity, which is "the degree to which a program is implemented as intended by its developers," (25) was addressed by several methods. Training materials were developed and used to standardize program delivery across group leaders. Group leaders participated in a mandatory half-day training session that provided an overview of the study goals, objectives, and content. Before each intervention session, group leaders received a scripted discussion guide for each session and handouts for study participants. Group leaders also met for about 2 hours with the principal investigator before each session to review and discuss session content and have any questions answered. During the meetings, the principal investigator modeled delivery of the session content and provided opportunities for the group leaders to provide input, ask questions, and practice delivering the intervention content. Intervention fidelity was also monitored by anonymous evaluation forms that participants completed at the end of each session and by examining weekly records of participant attendance and pedometer logs.

### Data collection

All measurements were collected at baseline and after 3 months except for records of daily walking, which were collected weekly throughout the study. All measurements were taken at the church where participants were recruited unless otherwise requested, and all measurements were taken by the same trained data collectors.

**Physical Activity Readiness Questionnaire.** The PAR-Q, which is a valid screening tool for pre-participation in PA (27-30), was used to screen study participants. The PAR-Q includes seven questions that assess the presence or absence of several known risk factors that preclude participation in MPA or VPA without consent from a medical professional. Participants who answered "yes" to any question on the PAR-Q were required to obtain medical clearance from a health care provider before being enrolled in the study.

**Daily walking.** Daily walking was measured throughout the study using an Accusplit Eagle pedometer. Previous studies have demonstrated the feasibility of the pedometer as an objective measure of PA and as a potential motivator to increase PA levels (31,32). All participants received a pedometer and instructions for wearing the pedometer and recording daily walking. Group leaders instructed participants to wear the pedometer on the waistband, in line with the outside of the knee cap, during all waking hours unless the participant was immersed in water. Participants were instructed to clear the pedometer count each morning and to record the total number of steps taken at the end of each day using a log sheet. The log sheets included space to record the time the monitor was put on in the morning, the time it was removed at the end of the day, the day and date of recording, and the total number of steps taken for the day. Log sheets were provided each week for recording the number of daily steps, and participants submitted the log sheets weekly for feedback on walking progress.

Baseline number of steps per day were based on the average number of steps per day during week 0. Data for week 4 were based on the average number of steps per day during weeks 1 to 4, and data for week 12 were based on the average steps per day during weeks 5 to 12. We performed separate analyses using only data collected for weeks 4 and 12. Results were comparable with data based on average number of steps per day across weeks (data not shown); thus, to maximize the sample size available for data analysis, we used the average number of steps per day across weeks. In addition, we analyzed steps per day categorically: less than 5000 steps per day (sedentary); 5000 to 7499 (low active); 7500 to 9999 (somewhat active), 10,000 to 12,499 (active), and 12,500 or more (highly active) (33).

**Moderate- and vigorous-intensity physical activity.** Self-reported participation by minutes per week in MPA, VPA, and walking was assessed using a modified version of the International Physical Activity Questionnaire (IPAQ) (34). The IPAQ assesses adherence to national recommendations for MPA and VPA during the previous 7 days. Two questions assess days per week and time per session for VPA or MPA lasting 10 minutes or more per session. Three questions assess daily and brisk walking at work, at home, for transportation, and for recreation, sport, exercise, or leisure for 10 minutes or more per session. To increase the accuracy of self-reported data, we modified the questions by changing open-ended questions related to frequency to closed-ended questions. Participants selected days per week from a list ranging from 0 days to 7 days. We also modified the questions on duration of PA from an open-ended response to categorical responses to try to reduce overestimation: "I do not do _____ activity for more than 10 minutes in a row"; "10 to 15 minutes"; "15 to 30 minutes"; "30 to 45 minutes"; "45 to 60 minutes"; and "Over 60 minutes." To calculate a continuous measure of PA participation, 0, 10, 15, 30, 45, and 60 were used to estimate minutes per day. We modified the question order from the original version of the IPAQ and asked about daily walking before asking about other MPA. We modified the timing to ask about usual PA instead of PA in the past 7 days. Minutes per week in MPA were calculated as the sum of days per week multiplied by minutes per day in MPA and brisk walking. Minutes per week in VPA were calculated as the sum of days per week multiplied by minutes per day in VPA.

**Participant characteristics and anthropometrics.** At the baseline visit, we used a questionnaire to measure self-perceived general health and well-being and presence of chronic diseases. Using a digital scale, we weighed each participant twice and measured a third time if the two measures differed by more than 0.2 kg. Height was also measured twice to the nearest 0.5 cm using a height stadiometer and measured a third time if the two measures differed by more than 0.5 cm. The average of the two closest measures was used for height and weight. Body mass index (BMI) was calculated as weight (kg) divided by height (m^2^). Seated resting blood pressure was measured, in duplicate, using a digital Omron monitor after participants had been seated with legs uncrossed for at least 5 minutes. Blood pressure was measured a third time if the two measures differed by more than 4 mm Hg. The two closest measures were averaged and used for analyses.

**Statistical analysis.** Statistical analyses were performed using SAS version 8.2 (SAS Institute Inc, Cary, North Carolina) and SAS-callable SUDAAN software (RTI International, Research Triangle Park, North Carolina). We examined the magnitude of the intra-class correlation coefficient (ICC) among subjects from the same church, which was estimated to be 0.06. We used a SUDAAN modeling procedure with two levels of clustering (both church and individuals specified in the models) to account for the ICC among church members and temporal dependence in repeated observations obtained on the same individual. Working independence between participants from the same church was assumed, along with an exchangeable covariance structure for repeated measurements on the same individual. Least square means were obtained from the models along with standard errors, which were then converted into standard deviations for the adjusted mean changes.

## Results

At baseline, participants (n = 87) were 52 ± 14 years of age (range, 20–83 years) with an average BMI in the obese category (34.7 ± 9.0 kg/m^2^; range, 16.1 to 63.3 kg/m^2^; 69% of participants had BMIs ≥30 kg/m^2^). All participants were black, most were female (89%), almost half (49%) were married, and most (96%) had a high school-level education or higher. Eighty-five percent of study participants reported at least one chronic health condition. Almost half (43%) of study participants reported using antihypertensive agents, and the mean systolic/diastolic blood pressure levels of study participants were in the borderline hypertension range (137 ± 23 mm Hg systolic and 84 ± 14 mm Hg diastolic). The four churches included in the intervention were similar across all baseline characteristics with the exception of education (*P* < .01).

Participants reported 26.6 ± 54.2 minutes per week in MPA and 10.0 ± 25.3 minutes per week in VPA at baseline. Consistent with self-reported participation in MPA and VPA, participants walked 4822 ± 2351 steps per day on average at baseline and met the definition for sedentary lifestyle (less than 5000 steps per day [33]). Most participants (57%) were in the sedentary category, although almost one-third (30%) were considered "low active." Very few participants were considered "somewhat active" and "active" (12% and 1%, respectively).

Data on mean changes in study outcome variables are presented in Table 2. Study participants attended 6.2 ± 1.8 of the eight intervention sessions. After four intervention sessions, steps per day increased by 846 ± 2047 steps (an 18% increase) to an average of 5729 ± 2230 steps per day (*P* = .04). The increase in steps per day was 1373 ± 728 steps (a 28% increase) after 12 weeks to an average of 6148 ± 2534 steps per day (*P* < .01). Although our sample size was not large enough to detect statistically significant differences, we observed a decrease in the proportion of participants who were categorized as sedentary (36% after 4 weeks, 32% after 12 weeks), and an increase in the proportion of participants who were classified as somewhat active (20% after 4 weeks, 28% after 12 weeks). There was an increase from baseline to 4 weeks in the proportion of participants who were classified as active (from 1% to 4%), but no additional change was observed after 12 weeks (4% classified as active). Participants also reported increases in self-reported minutes per week in MPA (66.9 ± 77.6 or 251%, *P* < .01) and VPA (43.8 ± 66.4 or 438%, *P* < .01). Weight and BMI remained stable over the 12-week intervention (data not shown). Changes in systolic and diastolic blood pressure after 3 months were in the expected direction (−4.3 ± 29.7 mm Hg and −3.4 ± 20.4 mm Hg, respectively) but were not statistically significant (data not shown).

## Discussion

We observed statistically significant increases in number of steps per day after 4 weeks and after 12 weeks, and significant changes in MPA and VPA after 12 weeks. We were encouraged to see that the proportion of participants who were classified as sedentary on the basis of previously published criteria for evaluating steps per day (33) decreased, and the proportion of participants who were classified as somewhat active and active increased. Although changes in systolic and diastolic blood pressure were not statistically significant, changes were in the expected direction. Weight and BMI remained stable over the 12-week intervention. This study adds to the existing literature on successful methods for increasing PA levels in black communities and provides preliminary information about the potential for a faith-based program to increase PA levels over a 3-month period among sedentary black adults.

Findings from our study regarding changes in PA among blacks are in line with, and in some cases show better results than, studies with similar time frames and study designs. An uncontrolled community-based walking program among 24 black breast-cancer survivors (aged 47 to 66 years) included eight 75-minute weekly sessions held at either a community center or a local church (35). The study used a curriculum that described the benefits of and barriers to exercise, the relationship between exercise and health and cancer risk, and personal assessment and problem-solving sessions for motivation. Data for number of steps per day were collected at baseline, 8 weeks, and 12 weeks. Findings showed significant increases in steps per day from baseline after 8 weeks, but no further changes after 12 weeks. Banks-Wallace and Conn conducted an uncontrolled trial evaluating a 12-month walking intervention with 6-month follow-up in a sample of 21 sedentary, hypertensive black women (aged 25 to 68 years) (36). The intervention included 3-hour monthly group meetings and a home-based walking component. For the subset of women who attended all data collection visits (n = 10), the number of steps per day increased by 51% after 6 months but decreased by 13% at the end of the 12-month intervention. An 8-week diet and exercise education program among 10 mothers with children aged 0 to 3 years showed significant decreases in resting heart rate, which suggested improvements in cardiovascular fitness (37). A 12-week study condicted among urban black middle-school children (n = 56) and their parents (n = 25) and designed to promote fruit and vegetable intake and increase exercise showed significant decreases in walk/run time for parents as well as significant decreases in BMI, body fat, and resting diastolic blood pressure (38).

Not surprisingly, we did not observe significant changes in blood pressure in our study. The pilot study had a large enough sample to test meaningful differences in daily walking as assessed by a pedometer but was not large enough to be able to detect small changes in blood pressure. We were encouraged, however, to see trends in the appropriate direction for these variables. We were also not surprised that weight did not change in the current study. Hill et al suggest that small changes in behavior, such as adding an extra 2000 to 2500 steps per day, may prevent excess weight gain (39). Changes in steps per day in our study approached but did not reach Hill's recommendation. Data from the National Weight Control Registry (NWCR) suggest that individuals who successfully maintain weight loss walk an average of 11,000 to 12,000 steps per day (40). Even though participants in our study increased daily walking over baseline levels, the average daily step count after 3 months was about 6100 steps per day, well below estimates from NWCR. To our knowledge, no studies have articulated the change in steps per day necessary for significant changes in clinical outcomes, and additional research is needed in this area.

Our study had a number of limitations. First, because we were unclear about the feasibility of the proposed study design and because of the reluctance in some faith-based communities to be part of a randomized study, particularly to a no-attention control group, we did not use a randomized, controlled design. We recognize the lack of a control group as a major limitation of the study. Second, this study's sample group was small; additional participants may have improved our ability to detect differences in clinical variables. Third, we did not have objective data for participation in MPA and VPA. We attempted to collect these data using accelerometers but encountered several issues related to adherence to the accelerometer data collection protocol and, thus, were not able to use these data for analyses. Finally, we are aware that participants who enrolled in our study might differ from the general population, from individuals who do not attend church, and from individuals who chose not to volunteer for the study.

The study also had several strengths. Although we were unable to obtain an objective measure of MPA and VPA, we were able to collect this information using a self-reported questionnaire. Although self-reported PA data tend to be overestimated, increases in MPA and VPA in our study were in line with increases in objectively measured PA. We addressed some limitations of previous studies by incorporating tenets of the church and by involving the church in intervention development. Participants reported high satisfaction with the intervention, suggesting that this strategy might be successful in other black churches. At least one church used the materials to conduct a second set of intervention classes with new participants. Although we did not collect outcome data on the new participants, anecdotal evidence suggests that the program was well accepted in the second set of classes. Group leaders were able to implement the program on their own, suggesting that the program strategy may be sustainable. Finally, the current pilot study found that participants increased their number of steps per day and approached the level suggested to prevent weight gain (39).

These data suggest that a faith-based PA intervention may be an appropriate strategy for increasing PA among sedentary black adults. Future research will determine the impact of this program compared with a control group. We plan to conduct a randomized, controlled trial to compare the faith-based PA intervention with a control condition.

## Figures and Tables

**Table 1:** Intervention Session Topics and Incentives, Faith-Based Physical Activity Intervention for Black Adults, North Carolina, March 2005–October 2005

Session	Session Content	Physical Activity	Incentive
1	Introduction	Stretching/audio CD	Pedometer
2	Goal setting; establishing a walking plan	Outside walk or exercise video	Audio exercise CD
3	Fitting physical activity in; time management	Seated aerobics	Tote bag
4	Problem solving and negotiating barriers	Outside walk or exercise video	Exercise video
5	Healthful living and Biblical perspectives	Video	Audio exercise CD
6	Developing a support network	Outside walk or exercise video	Exercise video
7	Incorporating other activities	Strength training	Strength-training bands
8	Restarting a suspended physical activity program	Outside walk or exercise video	T-shirt

**Table 2:** Mean Change Over Time in Physical Activity (PA)^a^, Faith-Based Physical Activity Intervention for Black Adults, North Carolina, March 2005–October 2005

Variable	Amount of Activity			Adjusted Mean ChangeFrom Baseline to Follow-up	

	No. of Participants	Time	Mean (SD)	Mean (SD)	*P* value
Steps walked/day	77	Baseline	4822 (2351)	NA	NA
	69	Week 4	5729 (2230)	846 (2047)	.04
	71	Week 12	6148 (2534)	1373 (728)	<.001
Moderate PA (min/wk)	87	Baseline	26.6 (54.2)	NA	NA
	71	Week 12	93.7 (83.6)	66.9 (77.6)	<.01
Vigorous PA (min/wk)	87	Baseline	10.0 (25.3)	NA	NA
	71	Week 12	53.7 (65.0)	43.8 (66.4)	<.01

a Adjusted for church
